# Progesterone receptor, prokineticin 1, LncRNA-H19, and miR-210 in molecular regulation of recurrent implantation failure

**DOI:** 10.1038/s41598-026-41482-7

**Published:** 2026-06-08

**Authors:** Rahim Rostami, Jamileh Jahanbakhsh, Shirin Salehi, Katayoon Asgari, Soudabeh Fallah

**Affiliations:** 1 Department of Clinical Biochemistry, Faculty of Medicine, Iran University of Medical Sciences, Shahid Hemmat Highway, PO-BOX: 1449614535, Tehran, Iran; 2 Infertility Ward, Arash Women’s Hospital, Tehran University of Medical Sciences, Tehran, Iran; 3 Department of Pharmacology, Faculty of Pharmacy, Iran University of Medical Sciences, Tehran, Iran

**Keywords:** Recurrent Implantation failure, Progesterone receptor, Prokineticin 1, lncRNA-H19, miR-210-5p, Biochemistry, Biomarkers, Medical research, Molecular medicine

## Abstract

Recurrent implantation failure (RIF) is a major challenge in reproductive medicine. This study explores the expression profiles and interactions of progesterone receptor B (PGR-B), prokineticin 1 (PROK1), prokineticin receptor 1 (PROKR1), and their regulatory associations with microRNA-210-5p (miR-210-5p) and long non-coding RNA H19 (lncRNA-H19) within endometrial tissue. This case-control study was conducted from June 2021 to July 2023, involving 50 patients with recurrent implantation failure (RIF) and a control group of 50 women with successful term pregnancies. Participants were recruited from Arash Hospital in Tehran, Iran. Hysteroscopic assessments were done for the RIF group, while the control group had sampling via Pipelle catheter. We measured the expression levels of key molecular markers, including PGR-B, PROK1, PROKR1, lncRNA-H19, and miR-210-5p, using quantitative real-time PCR (qRT-PCR). Our findings demonstrate significantly low expression and protein levels of PGR-B and PROK1, suggesting a potential dysregulation of these biomolecules in the studied context. Notably, we observed a strong positive correlation between PGR-B and PROKR1, suggesting a synergistic regulatory relationship, potentially influencing endometrial signaling pathways. Conversely, in fertile women, PGR-B and PROK1 exhibited a positive correlation with miR-210-5p, implicating miR-210-5p as a potential activator of PGR-B downstream pathways. Additionally, a positive correlation between PGR-B and PROK1 with lncRNA-H19 was identified, hinting at a complex regulatory network involving these non-coding RNAs. Our findings indicated that a regulatory network involving PGR‑B, PROK1, PROKR1, lncRNA‑H19, and miR‑210‑5p in women with RIF. The significant correlation between these molecules underscores the importance of angiogenesis, a novel mechanistic link to impaired decidualization and endometrial receptivity. It provides new insight into the molecular basis of implantation failure.

**Supplementary Information:**

The online version contains supplementary material available at 10.1038/s41598-026-41482-7.

## Introduction

Infertility affects almost 17% of couples globally. In vitro fertilization with embryo transfer (IVF-ET) has improved pregnancy success rates, although some experience recurrent implantation failure (RIF), defined as not conceiving after transferring four quality embryos in at least three cycles. RIF can be caused by embryo or maternal factors, including chromosomal abnormalities, maternal medical conditions, Uterine anatomical abnormalities, and immune factors^1^. Current studies report true RIF is extremely uncommon, occurring in < 5% of couples with infertility^2,3^.

Endometrial receptivity stands as the primary limitation during embryo implantation, making receptivity disruption the major cause of RIF. The intricate process of endometrial receptivity is governed by estrogen, progesterone and ovarian hormones, which regulate gene expression patterns in endometrial cells. These hormones induce distinct morphological and functional changes that support embryo attachment and implantation^4,5^.

Progesterone is essential for regulating receptivity by activating receptors that influence endometrial stromal and epithelial cell functions. During the mid-to-late secretory phase of the endometrium, the expression of progesterone receptors (PGR) in epithelial cells is low. Conversely, the stromal cells exhibit significantly higher expression of *PGR* throughout this period. Expression levels of *PGR* gene, PGR protein and phosphorylated PGR protein significantly reduced in women with RIF than fertile women without RIF. Successful implantation requires the presence of PGR, which relies on having a healthy embryo and a receptive endometrial environment^4–6^.

Prokineticins, a group of peptides with diverse roles in angiogenesis, reproduction, hematopoiesis, neuronal activity, and immune response, are classified into *PROK1 and PROK2*, a peptide composed of 86 amino acids, is a major player in the female reproductive processes, particularly in ovarian physiology, endometrial receptivity, and embryo implantation^7–9^. Its role in angiogenesis is so significant that it is also known as endocrine gland-derived vascular endothelial growth factor (*EG-VEGF*)^10^. Recent investigations have revealed that the expression of *PROK1* in reproductive tissues—including the ovary, uterus, and placenta—is modulated in response to shifts in the hormonal milieu. Variations in estrogen, progesterone, human chorionic gonadotropin (hCG), and hypoxia-inducible factor (*HIF-1α*) are significant throughout the menstrual cycle and are critical during pregnancy, particularly influencing placental development^11^. Research has indicated that proliferation-activating proteins (PROKs) are implicated in various pregnancy-related disorders, including endometriosis, RIF, polycystic ovary syndrome (PCOS), intrauterine growth restriction (IUGR), and choriocarcinoma. These conditions may stem from defects related to decidualization, implantation, and/or placentation failure^12–14^.

Long non-coding RNAs (lncRNAs) and miRNAs play a critical role in regulating fertility and infertility through gene expression modulation. miRNAs, as short non-coding RNAs, bind to target mRNAs to induce degradation or inhibit translation. In contrast, lncRNAs are longer and more versatile, acting as scaffolds and mediators in molecular interactions, significantly influencing gene transcription and epigenetic modifications. This complexity enhances our understanding of fertility mechanisms^15–19^.

Long non-coding RNA H19, one of the first identified lncRNAs, exhibits high expression levels during embryogenesis. Its downregulation has been linked to diminished invasion of extravillous trophoblast (EVT) cells, which plays a critical role in conditions such as intrauterine growth restriction and preeclampsia. Previous research has demonstrated that H19 is instrumental in modulating trophoblastic spheroid adhesion^20^. The processes of embryo implantation and placentation are multifaceted, involving intricate mechanisms of adhesion, invasion, and angiogenesis; any dysregulation within these pathways can precipitate implantation failure or miscarriage. Specifically, the downregulation of *H19* has been associated with a marked decrease in integrin β3 (*ITG-β3*) expression within the endometrial tissue and a reduction in *HOXA10*, an essential factor for endometrial receptivity^20–23^.

MicroRNAs (miRNAs) are small, non-coding RNAs (19–25 nucleotides) that regulate cell fate, function, and tumorigenesis. Abnormal miRNA expression has been linked to tumor progression and the epithelial-mesenchymal transition. Studies show that long non-coding RNA H19 (lncRNA-H19) interacts with miRNAs in endometrium to improve proliferation and receptivity. lncRNA-H19 and miRNA interaction is key factor in regulation of angiogenic related genes and regulation of endometrial receptive^24^. However, the relationship between lncRNA-H19 and miRNAs and PROK1/PGR-B expression in women with recurrent implantation is still unclear.

The current literature reveals a notable gap concerning the involvement of PROKs in the etiology and progression of RIF. Additionally, the interplay between steroid receptors and the modulation of *PROK1* expression has not been thoroughly characterized. We hypothesized that impaired expression of PGR-B, PROK1, and PROKR1, regulated by lncRNA-H19 and miR-210-5p, contributes to defective endometrial receptivity in RIF. This study aims to quantify the differential expression of PROK1, its receptor (PROKR1), PGR-B, and the lncRNA-H19-miR-210-5p axis in endometrial tissues from women with recurrent implantation failure (RIF) compared to age-matched fertile controls, using qRT-PCR and western blot analysis. The research aims to investigate the regulatory interactions between PGR-B and PROK1 at both the transcriptional and protein levels, emphasizing the functions of lncRNA-H19 and miR-210-5p as modulators at the post-transcriptional stage. Furthermore, it intends to link these molecular interactions to the mechanisms that contribute to embryo implantation failure in instances of recurrent implantation failure (RIF).

## Materials and methods

This study was conducted from June 2021 to December 2023 and in accordance with the ethical standards of the Iran University of Medical Sciences Ethics Committee and the 1964 Declaration of Helsinki and its later amendments. The study protocol was approved by the Iran University of Medical Sciences Ethics Committee (approval number: IR.IUMS.FMD.REC.1402.040). All experimental procedures were performed in accordance with the relevant guidelines and regulations, and written informed consent was obtained from all participants prior to inclusion. Current studies report that 2–5% of women experience recurrent implantation failure after more than four high-quality embryo transfers^2,3^. Our study sample consisted of 100 subjects (*n* = 50 RIF and *n* = 50 Control). The sample size was calculated using the Chi-square test, assuming a statistical power of 80% and a significance level of 0.05.

### Inclusion and exclusion criteria

The study centered on a **case-control study** of 50 female patients under the age of 42 who were referred to the Medical Center of Referral Arash Hospital. These individuals had undergone IVF/ICSI treatment and had encountered a minimum of three unsuccessful fetal transmissions, despite receiving four or more high-grade morphological embryos. Additionally, a control group of 50 women who had no prior IVF history and were experiencing secondary infertility due to male infertility, tubal factors, or unexplained infertility was included. The control group had previously experienced successful term pregnancies with live births.

Exclusion criteria include: poor embryo quality and ovarian response, known disorders of the uterus or endometrial pathologies, thrombophilias, diabetes and thyroid diseases, PCOS, intrauterine pathologies, adenomyosis, endometriosis, and women/men with positive anti-lupus anticoagulant, abnormal chromosomal karyotypes or miscarriage, endocrine disorders, infectious diseases, and users of contraceptives. To meet the inclusion criteria, both the patient and control groups were required to have regular ovulation periods (28–32 days) and normal endocrine profiles. In both groups, at least four high-quality embryos were obtained 48 h after oocyte retrieval and transferred according to IVF/ICSI protocols. This ensured that embryo quality was not a confounding factor, and implantation outcome depended primarily on endometrial receptivity.

### Sampling and treatment protocol

According to our previous study^25^, endometrial tissue samples were collected by hysteroscopy and Pipelle catheter from women with RIF and healthy women as controls, respectively. Serum sampling was also performed within 5 to 7 days after ovulation in all subjects. It is worth noting that the intrauterine pathologies commonly missed by other investigative modalities could be detected by hysteroscopy.

Ovulation time was determined by assessing the morning luteinizing hormone (LH) and transvaginal ultrasound (PHILLIPS AFINITY 70). The window period was defined as LH + 7 days. The day of the LH surge was LH 0. On this day (LH 0), endometrial tissue and serum samples were sent to the laboratory immediately after sampling and frozen at -80 °C for total RNA extraction. According to local protocols, IVF/ICSI was performed. All subjects had indications for IVF/ICSI treatment and underwent routine fertility tests and ovarian hyperstimulation with recombinant follicular-stimulating hormone (FSH) (Cinnal-F, Cinnal-F, Iran) or human menopausal gonadotropin (HMG) (Menotropin, Poish Darou, Iran). To prevent LH surge, the pituitary gland was suppressed using a gonadotropin-releasing hormone (GnRH) antagonist (Cetronax, Ronak, Iran / Cetrotide, Merck Serono, Germany). Final follicle maturation was triggered using 10,000 international units of human chorionic gonadotropin (HCG) (Gonarx, Ronak, Iran). Oocyte retrieval was performed 36 h after HCG injection through guided ultrasound. One and two fresh blastocyst embryos (grade A) were transferred by catheter (Rada, Behrad, Iran) to the control and RIF groups. Intravaginal transfusion of progesterone 800 mg/day (Fertigest, Aburayhan, Iran) was used to support the luteal phase in both groups.

### RNA Extraction and cDNA synthesis

Total RNA was isolated from endometrial tissue using a column-based extraction kit (Yekta Tajhiz, Tehran, Iran) according to established protocols, including homogenization, binding, washing, and elution. RNA purity and integrity were assessed by spectrophotometric measurement of the A260/A280 ratio and agarose gel electrophoresis. Complementary DNA (cDNA) for both mRNA and lncRNA targets was synthesized under standardized conditions using random hexamers and oligo(dT) primers.

### RNA quality assessment and RT-PCR

The concentration and purity of the extracted RNA were evaluated using a NanoDrop One spectrophotometer (Thermo Scientific, USA) and 3% agarose gel electrophoresis. Complementary DNA (cDNA) was synthesized from 1 µg of total RNA using a reverse transcription kit (cDNA synthesis premix, Zist Virayesh, Tehran, Iran). Endometrial gene expression was quantified using Real-Time Polymerase Chain Reaction (RT-PCR) on an ABI Step-One device (Applied Biosystems, USA). The reaction mixture (cDNA and master mix) was incubated in a 96-well plate at 95 °C for 10 min, followed by 40 cycles of 95 °C for 15 s and 60 °C for 40 s. Expression levels of PROK1, PROKR1, and PGR-B genes were calculated using the 2^−ΔΔCt method and normalized to GAPDH expression. All samples were processed in duplicate. For qRT-PCR, reactions were conducted in duplicate, with Ct values **showing < 5%** intra-assay variability. Inter-assay variability was minimized by running all samples for a given target gene under identical conditions, utilizing the same reagent batches, and normalizing to GAPDH/U6 controls.

Samples were coded at the time of collection, and personnel performing RNA extraction, qPCR, and Western blot analyses were blinded to group allocation. Data acquisition and statistical analysis were conducted using coded identifiers, and the sample processing order was randomized to minimize batch effects.

### miRNA amplification

Stem-loop RT-qPCR was conducted for miRNA amplification using the method described by Kramer et al.^26^. The stem-loop reverse transcription (RT) primer featured a modified 52-nucleotide sequence. This primer structure includes a stem region, a loop sequence, and a short miRNA-specific region at the 3′ end that is complementary to the last six nucleotides of the target miRNA. The stem-loop configuration is designed to enhance the specificity and efficiency of reverse transcription by minimizing nonspecific binding and distinguishing mature miRNAs from their precursor forms.

The stem-loop sequence is as follows:

$${\mathrm{5}}{\prime }{\text{ - CGTTGGCTCTGGTGCTGGGTCCGAGGTATTCGCACCAGAGCCAACGTCAGCC - 3}}{\prime }.$$

Specific forward and reverse primers targeting mature hsa-miR-210-5p were designed and synthesized for quantitative real-time PCR analysis. U6 small nuclear RNA was used as the endogenous reference gene to normalize miRNA expression levels.

The primer sequences are listed in Table 1.

**Table 1:** primers were used in this study.

Gene names	Primers
PGR-B gene	Forward: 5′-TCTACCCGCCCTATCTCAACTACC-3′ Reverse: 5′-TGTGCTGCCCTTCCATTGCC-3′
PROK1 gene	Forward: 5′-TGAGCGGGAGGAAGCGAGAG-3′ Revers: 5′-CCACACTGGACATCCCGCTC-3′
PROKR1 gene	Forward: 5′-AGATGGACTACTATGTGGTGCG-3′ Revers: 5′-TCTCAGCGGATGGACAATAGC-3′
lncRNA-H19	Forward: 5′-TGACGGCGAGGACAGAGGA-3′ Revers: 5′-ATGTTGTGGGTTCTGGGAGCC-3′
hsa-miR-210-5p	Forward: 5′- TCCATCCTGTGCGTGTGACAGC − 3′ Reverse: 5′- CGTTGGCTCTGGTGCTGGGT − 3′
GAPDH gene	Forward: 5’-CCCCTTCATTGACCTCAACTAC- 3’, Reverse: 5’-GATGACAAGCTTCCCGTTCTC-3′
U6	Forward: 5’- TTGGAACGATACGGAGAAGATTAGC- ‘3 Reverse: 5’-TATGGAACGCTTCACGAATTTGC- ‘3

### Protein western blot

To extract cellular proteins from homogenized endometrial tissue samples from women with RIF and controls, we employed a modified RIPA lysis buffer, based on the protocol from Thermo Fisher Scientific, supplemented with a protease inhibitor cocktail (Biobasic, Canada). Protein concentration was subsequently quantified using the Bradford assay.

For Western blot analysis, 12 µL of the extracted protein samples were subjected to SDS-PAGE for electrophoretic separation. Following this, proteins were transferred to a PVDF membrane (Millipore, USA) via electroblotting at 4 °C for 4 h. The membranes were then blocked using a buffer composed of 1% (w/v) gelatin in 50 mM Tris-HCl (pH 7.4) and 150 mM NaCl, incubated for 1 h at room temperature, and washed three times (5 min each) with Tris-NaCl buffer. Overnight incubation at 4 °C was performed using primary antibodies: a rabbit monoclonal IgG1 against PROK1 (Abcam, Cat No: ab248951, Cambridge, UK) and a mouse IgG1κ directed against PGR-B (Santa Cruz, Cat No: B-30, Dallas, Texas, USA). Afterward, membranes were washed three times with Tris-NaCl/Tween 20 (0.05%) and subsequently incubated with secondary antibodies: m-IgGκ BP-HRP and mouse anti-rabbit IgG-HRP (Santa Cruz, Dallas, Texas, USA) for 2 h at room temperature. Protein detection was performed using ECL Western blotting substrate, and images were captured using a Western blot imaging system. Band intensities were quantified using ImageJ software. β-actin (Santa Cruz, Cat. No. (2A3): sc-517582) served as the loading control, with all target protein intensities normalized to β-actin levels. Densitometric analysis was conducted using ImageJ software (NIH, USA), and relative expression values were averaged across independent experiments. The quantified data are presented as mean ± SD. Western blot analyses were performed in two independent experiments, with replicate variability **< 10% CV**, consistent with accepted reproducibility standards.

### Statistical analysis

Data analysis was conducted utilizing IBM SPSS Statistics version 24.0. Continuous variables are presented as mean ± standard deviation (SD) for data adhering to a normal distribution, and as median (min–max) for data exhibiting non-normal distribution characteristics. Normality was evaluated through the Kolmogorov–Smirnov test, while the Shapiro–Wilk test was employed for smaller sample sizes. When confronted with skewed distributions, a log transformation or other suitable methods were applied to facilitate approximation of normality prior to conducting parametric tests. For comparative analysis of continuous variables, Student’s t-test was utilized for normally distributed data, whereas the Mann–Whitney U-test was employed for nonparametric data. Categorical variables underwent analysis using the chi-square (χ²) test. Correlations among miRNA, lncRNA, and gene expression levels were analyzed using Pearson’s correlation for parametric datasets and Spearman’s rank correlation for nonparametric datasets. A two-sided p-value threshold of < 0.05 was established to denote statistical significance. Graphical representations in the form of box-and-whisker plots were generated using GraphPad Prism version 10.3.0 for Windows (GraphPad Software, La Jolla, CA, USA).

(For comparisons of multiple genes and miRNAs, raw p-values were adjusted for multiple testing using **Bonferroni and Benjamini–Hochberg (FDR) corrections**. Adjusted p-values are reported alongside raw p-values, and significance was defined as FDR < 0.05. Normality of data distribution was assessed using the Kolmogorov–Smirnov test. Variables that exhibited a normal distribution (miR-210, PGR-B protein, PROK1 protein) are presented as mean ± standard deviation (SD) and were analyzed using the independent-samples t-test. Variables with a non-normal distribution (PGR-B, PROK1, PROKR1, lncRNA-H19) are presented as median (min–max) and analyzed using the Mann–Whitney U test.)

## Results

As shown in Table 2, the demographic and hormonal characteristics of the recurrent implantation failure (RIF) group and fertile controls were largely comparable. No significant differences were observed in age, body mass index (BMI), serum progesterone (P4), estrogen (E2), or follicle-stimulating hormone (FSH) levels between the two groups. In contrast, luteinizing hormone (LH) concentrations were significantly reduced in RIF patients compared with fertile women (5.39 ± 4.41 vs. 12.45 ± 11.47 IU/L, *p* < 0.0001), highlighting a potential role of impaired LH regulation in the pathophysiology of RIF.

**Table 2:** The clinical characteristics of the study participants are presented in Table 2. The RIF and controls were similar in terms of age, body mass index, and levels of progesterone (P4) and estrogen (E2).

Parameters	RIF patients	Fertile women	*P*-Value
Age	37.32 ± 3.68 (39; 28–42)	36.68 ± 3.96 (36; 25–42)	0.405^a^
BMI (Kg/m^2^)	27.39 ± 2.97 (26.95; 22.31–33.45 )	26.54 ± 2.40 (25.56; 21.93–32.49)	0.116^b^
P4 (ng/mL)	9.33 ± 7.36 (10.1; 0.4–24.1)	14.81 ± 22.28 (11.4; 0.7–99.9)	0.102^b^
E2 (pg/ml)	94.34 ± 41.93 (88; 37–201)	90.86 ± 40.31 (95; 33.1–201)	0.637^b^
LH (IU/L)	5.39 ± 4.41 (3.40; 0.7–21.5)	12.45 ± 11.47 (8.85; 1.2–53.1)	< 0.0001^b^
FSH (IU/L)	4.19 ± 0.75 (4.0; 2.6–5.6)	4.09 ± 1.89 (3.55; 1.5–8.4)	0.719^a^

^a^Independent sample t-test,^b^Mann-Whitney U test, Mean ± SD, Median (Min-Max). Demographic and clinical characteristics of the study groups. Continuous variables were reported as mean ± SD when normally distributed (Age and FSH) and as median (min–max) when non-normally distributed (BMI, E2, P4, and LH), based on Kolmogorov–Smirnov and Shapiro–Wilk tests.

### Genes and proteins expression

**Fig. 1:**
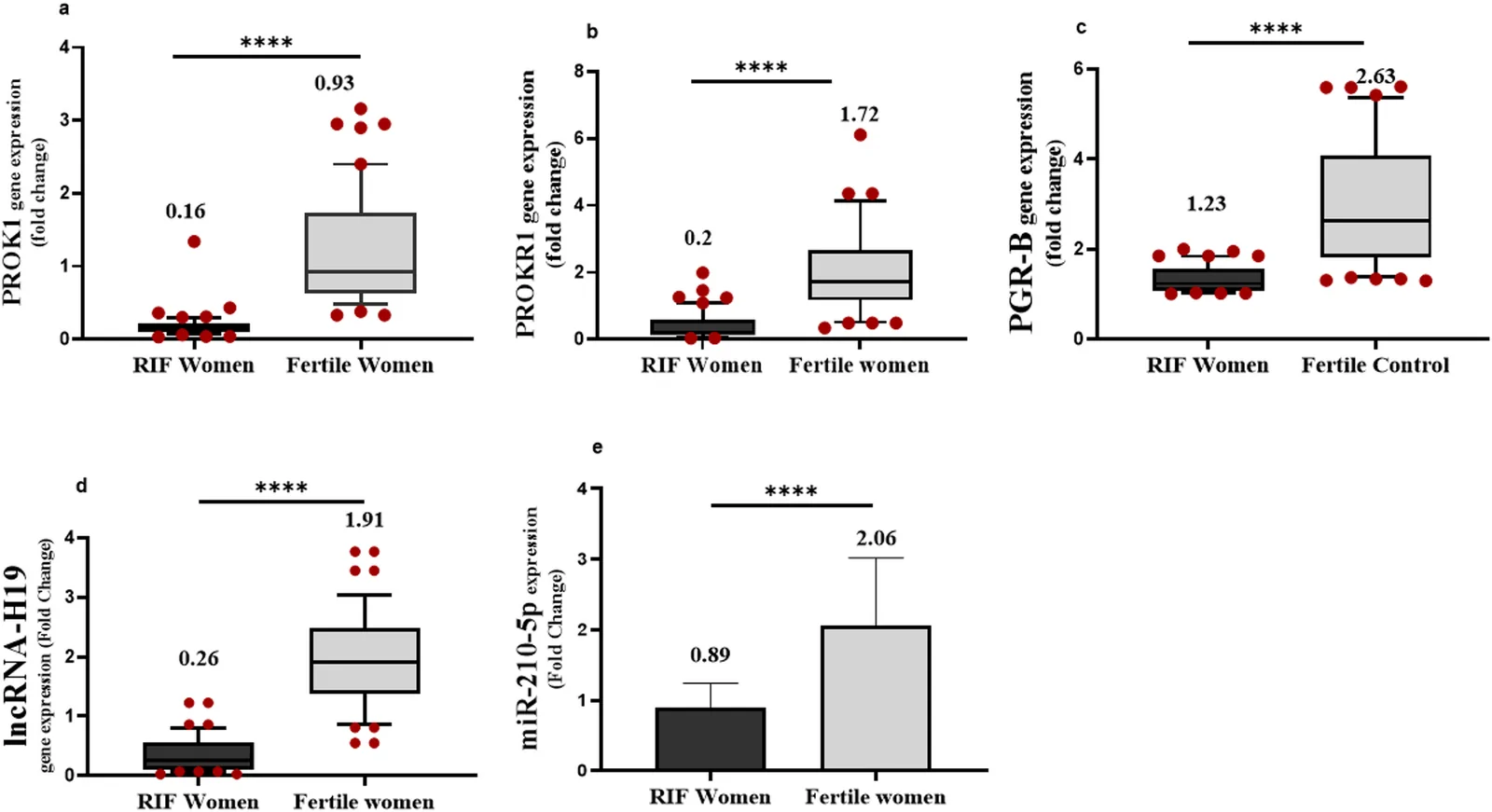
The endometrial expression levels of *PGR-B*,* PROKR1*,* lncRNA-H19*,* miR-210-5p* genes in RIF and fertile women without RIF. *Mann-Whitney U test were used for** a**-**d** and T-test used for figure **e**. All p-values were adjusted for multiple comparisons using the Benjamini–Hochberg procedure (FDR) and Bonferroni correction. Genes remaining significant after FDR adjustment are indicated (Sig_FDR = 1). **** *p* < 0.0001, Whisker plots, Min-Max (10–90 percentile). *** adjusted p-value < 0.001.

A statistically significant difference was observed in *PGR-B*, *PROK1*, and *PROKR1* expression levels between women with RIF and fertile controls (P-value < 0.0001 and adj p-value < 0.001). Specifically, relative mRNA expression levels of *PGR-B*,* PROK1*, and *PROKR1* were found to be reduced by 1.5, 2.0, and 2.5 times, respectively, in the endometrial tissue of women with RIF compared to that of fertile controls (Fig. 1). This indicates a marked reduction in the expression of these genes in the RIF cohort compared to the fertile group.

In patients with RIF, miR-210-5p and lncRNA-H19 were significantly downregulated in endometrial tissue compared with control groups (all *P* < 0.001; see Fig. 1). The expression of miR-210-5p was normally distributed, and the Mean ± SD was 0.89 ± 0.34 with 95%CI: 0.79–0.95 for RIF women. In fertile women, the mean ± SD was 2.06 ± 0.96, with a 95% CI of 1.79 − 2.33. The expression of lncRNA-H19 was non-parametric and therefore expressed in median (Min-Max) and box plot. The median (Min-Max) of lncRNA-H19 in RIF women was 0.26 (0.03–1.23) with 95%CI: 0.13–0.47, and for fertile women was 1.91 (0.55–3.77) with 95%CI: 1.45–2.25.

**Fig. 2:**
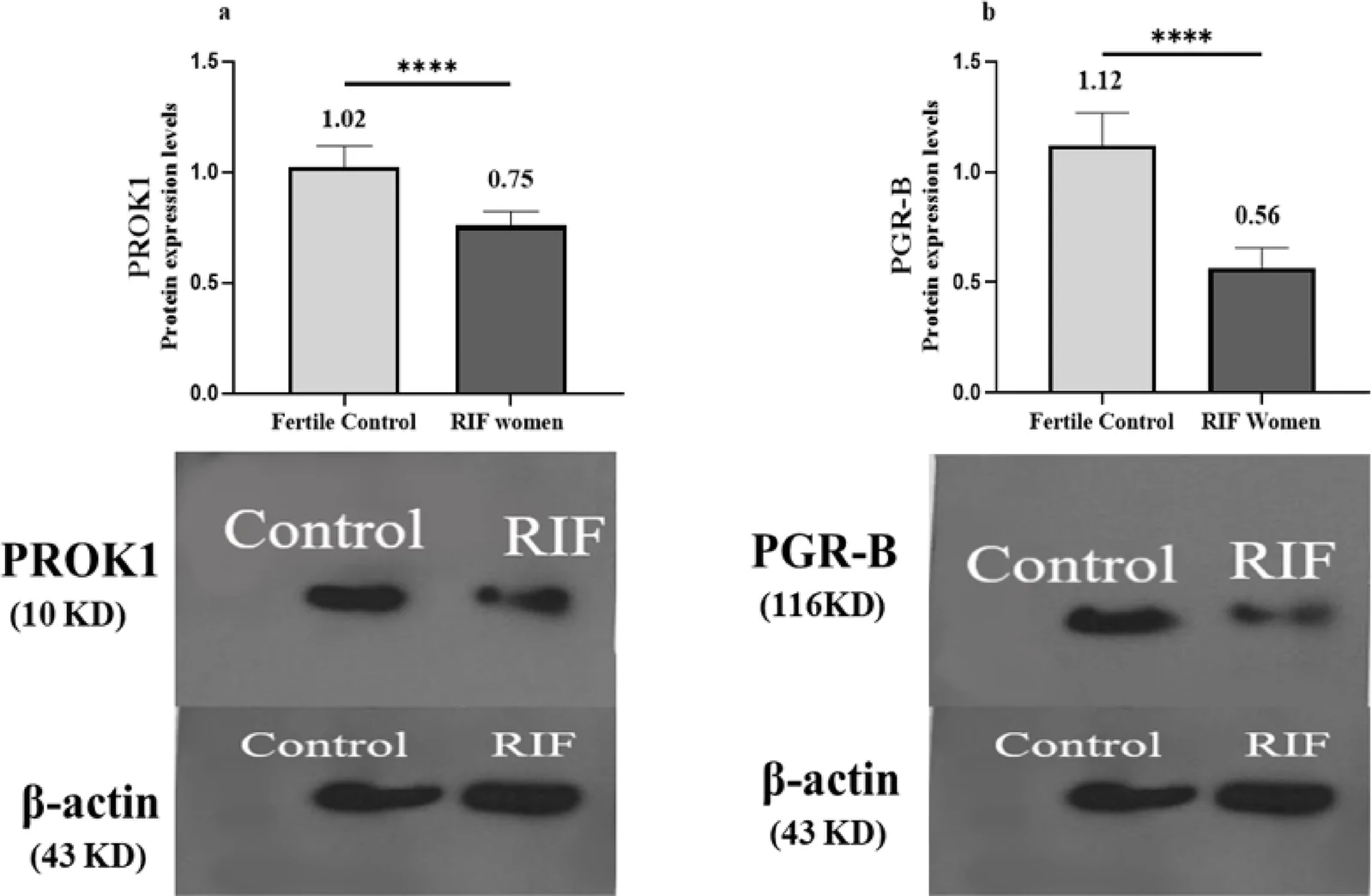
Protein expression levels of PGR-B and PROK-1 and western blot in RIF and fertile women. Western blot images and quantitative analysis of PGR-B and PROK1 protein expression in endometrial tissues from women with recurrent implantation failure (RIF) and fertile controls are presented. Protein levels were normalized to β-actin as the internal loading control. Data are shown as mean ± SD from three independent experiments. Statistical analysis was performed using Student’s t-test; **** *p* < 0.0001 and adjusted p-value < 0.001.

Additionally, Western blot analysis demonstrated markedly decreased protein levels of both PGR-B and PROK1 in the endometrial tissues of RIF patients relative to fertile counterparts (all *P* < 0.001) (Fig. 2a and b). For PROK1, the mean ± SD (95%CI) of protein expression was 0.75 ± 0.06 (0.73–0.77) for RIF patients and 1.02 ± 0.06 (0.99–1.07) for fertile women. For PGR-B, the mean ± SD (95% CI) of protein expression was 0.56 ± 0.09 (0.54–0.59) for RIF patients and 1.12 ± 0.14 (1.08–1.16) for fertile women.

### Correlations

**Fig. 3:**
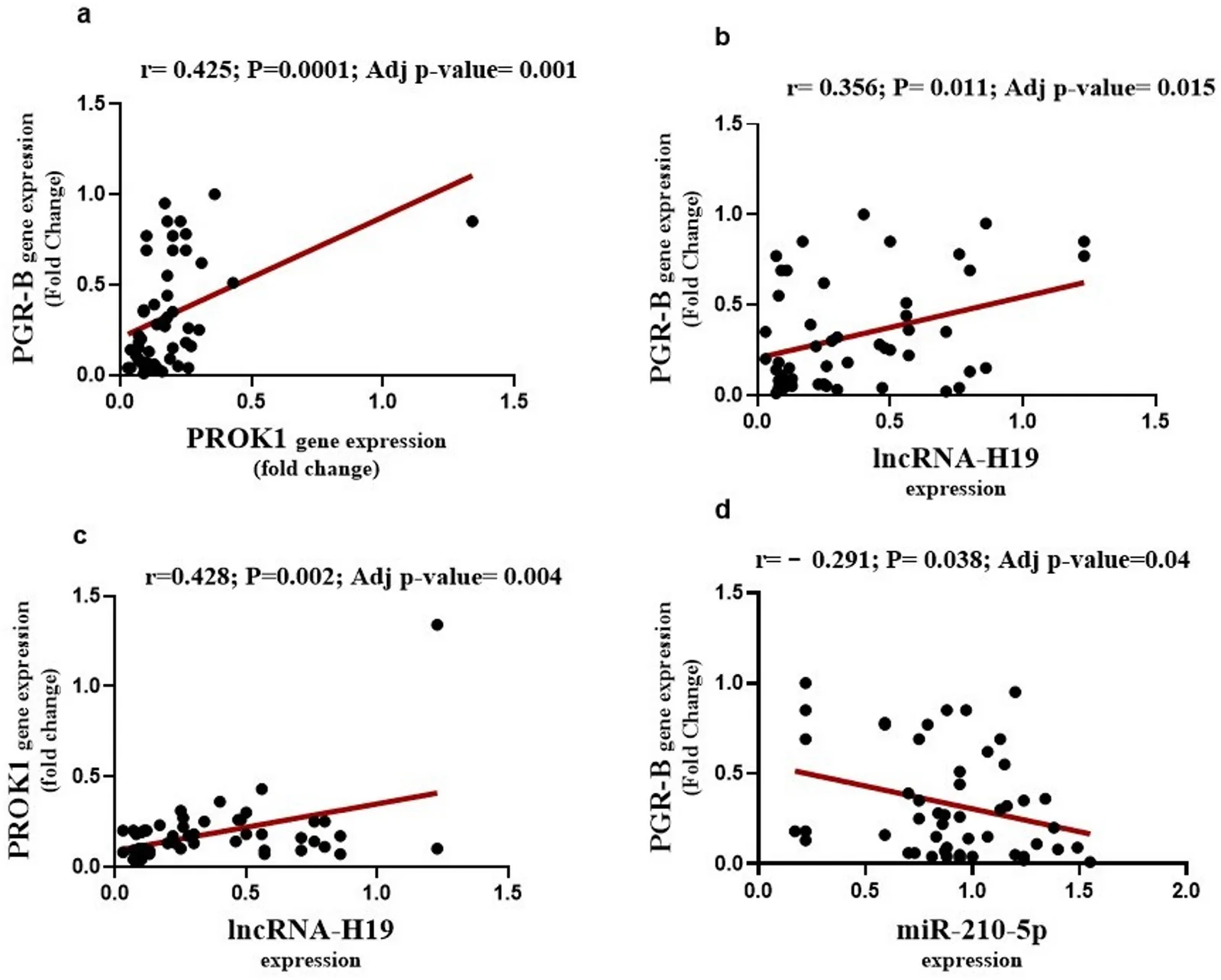
Scatter plots showing significant correlations between PGR-B and PROK1 expression, as well as between lncRNA-H19 with PGR-B and PROK1 expression levels in RIF patients. * Spearman’s rank correlation coefficient.

In women with RIF, significant positive correlations were found between *PGR-B* and *PROK1* gene expression, whereas an inverse significant association was observed between miR-210-5p with PGR-B gene (*r*= ‒ 0.293; *P* = 0.039 and adjusted p-value = 0.04). Moreover, it was found significant correlation between lncRNA-H19 with *PGR-B* and *PROK1* gene (Fig. 3a-d). However, no significant correlation was detected between gene expression, protein levels with lncRNA-H19 and miR-210-5p in RIF women.

**Fig. 4:**
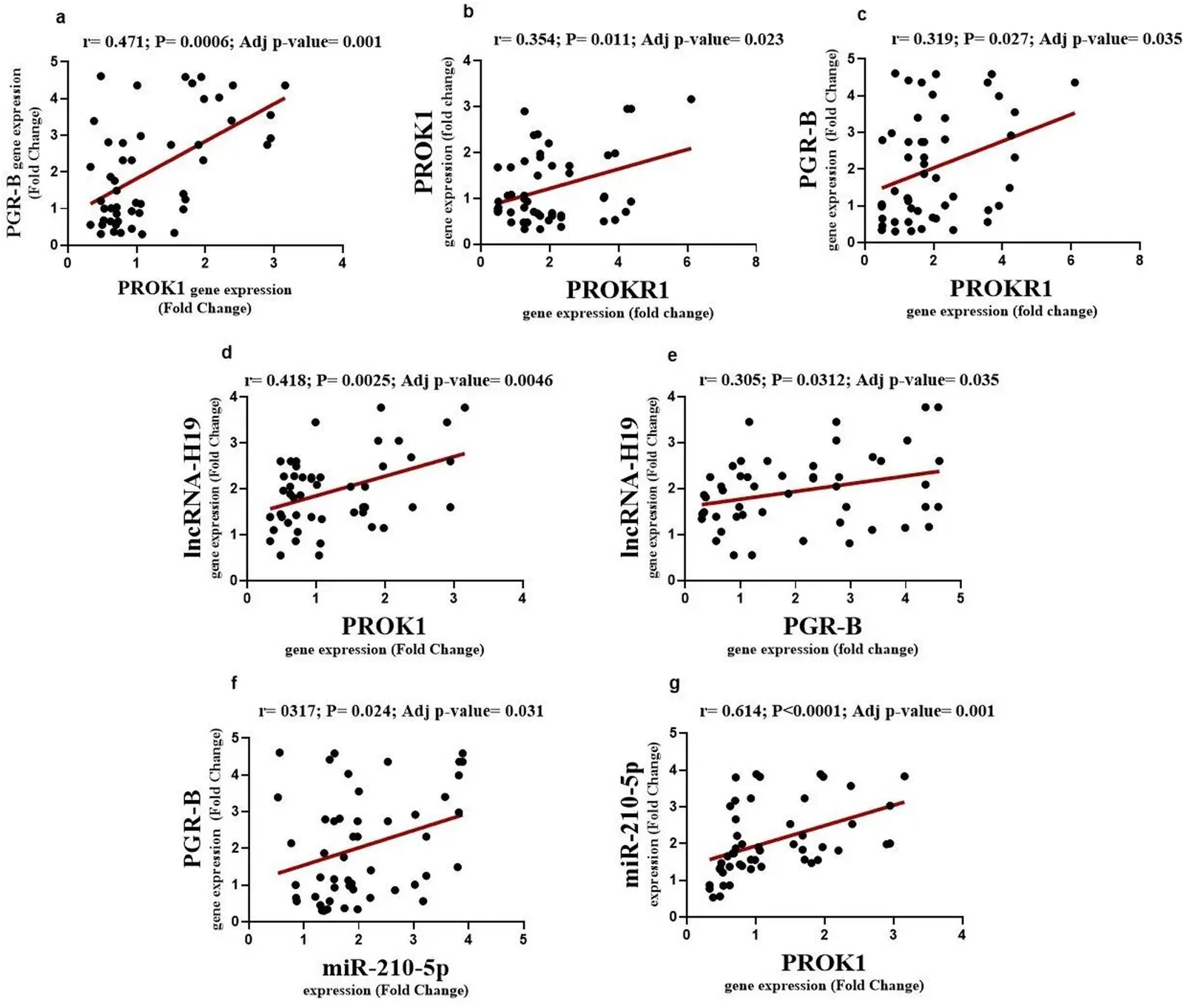
Correlation analysis showing significant associations between the expression levels of PGR-B, PROK1 and PROKR1 with lncRNA-H19 and miR-210-5p in fertile women. *Spearman’s rank correlation coefficient.

In fertile women, a significant positive correlation was observed between the *PGR-B* with *PROK1* and *PROKR1* (*P* < 0.0001; Adj p-value = 0.001 and *P* = 0.011; Adj p-value = 0.023 and *P* = 0.027; Adj p-value = 0.035, respectively (Fig. 4a-c). Additionally, a significant positive association was identified between *lncRNA-H19* with *PROK1and PGR-B* (*r* = 0.418; *P* = 0.0006; Adj p-value = 0.001 and *r* = 0.305; *P* = 0.0312; Adj p-value = 0.035) (Fig. 4d-e). Furthermore, *miR-210-5p* exhibited a positive association with the expression levels of *PGR-B* and *PROK1* which are presented in Fig. 4f-g (*r* = 0.317; *P* = 0.024; Adj p-value = 0.031 and *r* = 0.614; *P* < 0.0001; Adj p-value = 0.001). However, no significant correlation was found between *lncRNA-H19* and *miR-210-5p.* For all figures, the adjusted p-value is calculated and included in the table alongside the original p-value.

**Fig. 5:**
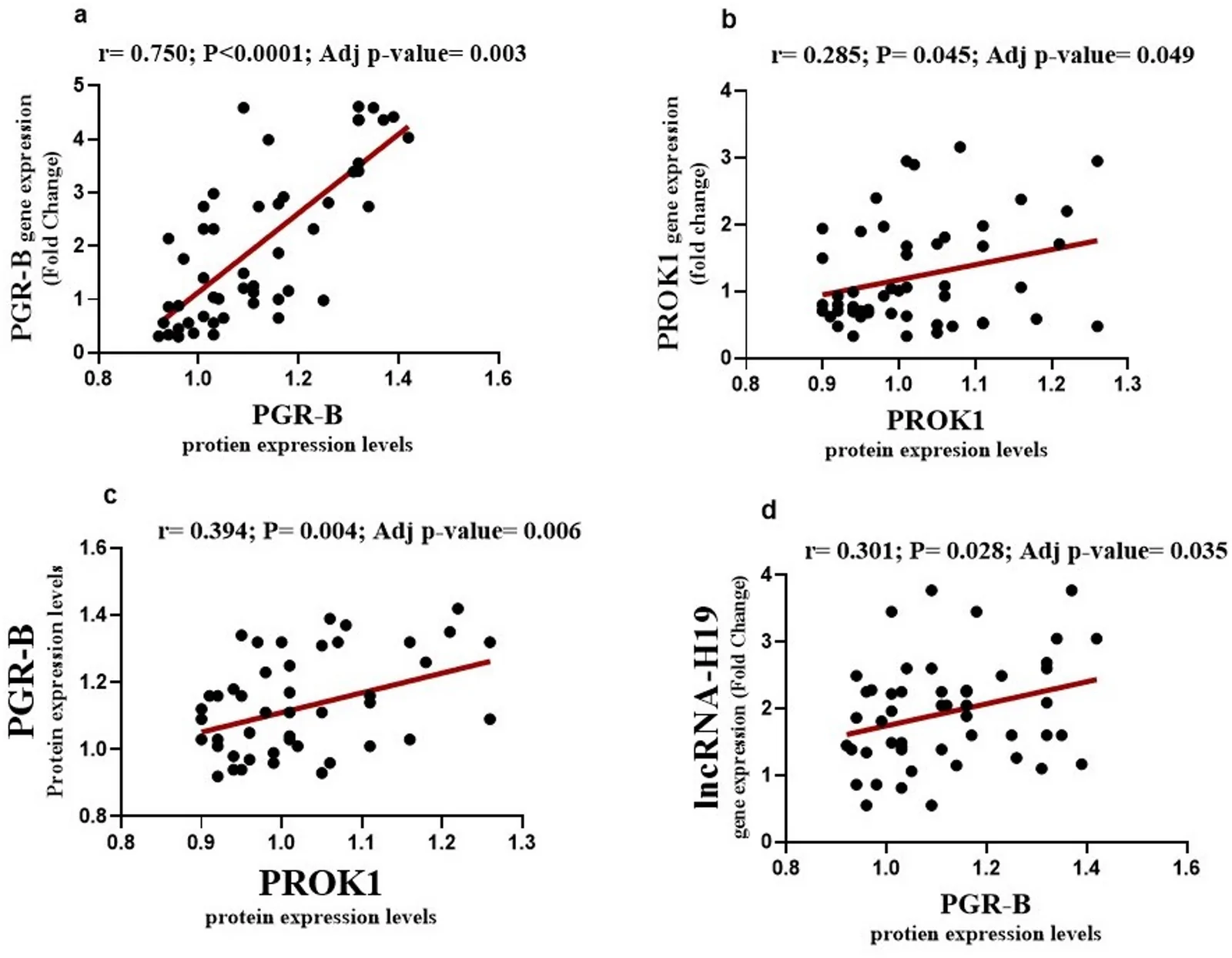
Scatter plots illustrating significant correlations between PGR-B and PROK1 protein expression levels and between their respective gene and protein expression levels in fertile women. *Spearman’s rank correlation coefficient.

In fertile women, protein expression levels are significantly associated with gene expression levels. PGR-B and PROK1 protein expression levels were significantly associated with *PGR-B* and *PROK1* gene expression (*r* = 0.748, *P* < 0.0001, and *r* = 0.285, *P* = 0.045, respectively). Also, there was a significant positive association between PROK1 and PGR-B protein expression levels (*r* = 0.394, *P* = 0.005) (Fig. 5a-c). There was a positive and significant correlation between lncRNA-H19 and PGR-B protein expression levels (*r* = 0.303; *P* = 0.030) **(**Fig. 5d). However, no significant association was found between lncRNA-H19 and miR-210-5p with protein levels. In fertile females, correlation analysis demonstrated a robust positive association between PGR-B gene expression and PGR protein levels (*r* = 0.750). This relationship maintained a high level of significance even after adjustment (adjusted p-value = 0.003). A moderate correlation was identified between PGR-B and PROK1 protein expression levels (*r* = 0.394), yielding a significant adjusted p-value (adj p-value = 0.006). Conversely, the correlation between PROK1 gene expression and PROK1 protein levels was weaker (*r* = 0.285), yet still significant after adjustment (adj p-value = 0.049). Also, a weak but significant correlation was found between lncRNA-H19 and PGR-B protein expression levels, even after adjustment (adj p-value = 0.035).

Positive correlations were observed among PGR-B, PROK1, lncRNA-H19, and miR-210-5p expression levels, suggesting a possible regulatory association. However, these findings should be interpreted as correlative rather than causal, as no functional assays were performed to validate direct molecular interactions. Significant positive correlations were found between PGR-B, PROK1, lncRNA-H19, and miR-210-5p in fertile women, indicating a coordinated regulatory network. In women with RIF, associations were observed as the same parameters, whereas a negative association was detected between PGR-B and miR-210-5p, which may indicate a disruption in the regulatory axis. All reported associations maintained statistical significance after FDR correction, suggesting the reliability of these findings.

## Discussion

Early pregnancy in mammals relies on the intricate crosstalk between the embryo and the uterine environment, predominantly modulated by hormones and growth factors. A critical component of this communication is angiogenesis, the process of new blood vessel formation, which is vital for establishing a supportive interface between the embryo and the maternal tissue. In this regard, Prokineticin 1 (PROK1) and its receptor, PROKR1, play pivotal roles in facilitating angiogenic processes within the endometrium, thereby enhancing embryo-maternal communication in both animal models and humans. Our study reveals that women experiencing RIF exhibit marked downregulation of PGR-B, PROK1, and PROKR1 in endometrial tissue, as demonstrated at both mRNA and protein levels. Additionally, we observed downregulation of lncRNA-H19 and miR-210-5p in RIF patients when compared to their fertile counterparts. Notably, a strong correlation between gene expression and protein levels was identified in the endometrium of fertile women, underscoring the significance of these molecular players in the successful establishment of implantation and early pregnancy.

Current knowledge on the expression of the PROK1/PROKR system in human endometrial receptivity and folliculogenesis remains scarce. Our recent investigation revealed significant alterations in the expression profile of PROK1/PROKR1 in the endometrium of women with recurrent implantation failure (RIF). Notably, we found that PROK1/PROKR1 expression was downregulated in patients diagnosed with RIF. During the implantation window, PROK1 expression in the endometrial tissue peaks and is localized in glandular epithelium, stromal, and endothelial cells. Multiple studies have highlighted the significant role of PROK1 in successful embryo implantation. The PROK1/PROKR1 signaling system also regulates the expression of key genes (such as LIF, COX-2, and Dickkopf-1) crucial for implantation processes^7,9,27^. In addition, during early pregnancy, the expression of PROK1 and PROKR1 genes is elevated in the human decidua compared to the non-pregnant endometrium, and this expression is regulated by human chorionic gonadotrophin (hCG), E2, and P4^12^. In particular, Alfaidy et al.^28^. investigated the expression and localization of PROK1/PROKR proteins in ovarian follicles at distinct developmental stages. Their study revealed a dynamic expression profile throughout folliculogenesis, characterized by pronounced staining in primordial and primary follicles, whereas expression was reduced in antral follicles. These results are consistent with earlier research on the expression of PROK1 transcripts during follicular development. Moreover, they found that the levels of PROK1 in follicular fluid and fertilization culture media were significantly higher in the embryo implantation group (IVF)^28^.

In a study by Goryszewska-Szczurek et al.^29^, it was found that PROK1 mRNA was elevated in porcine trophoblasts during implantation and the early placentation period. The study demonstrated that the PROK1-PROKR1 signaling pathways activate the transcription of genes involved in angiogenesis, trophoblast cell adhesion, invasion, immune response, and cellular proliferation by promoting the phosphorylation of MAPK and PTK2. Additionally, the study suggested that increased PROK1 expression in porcine 12 days of pregnancy could enhance the chance of clinical pregnancy through two biological pathways. In the first pathway, PROK1 could increase progesterone production, and in the second pathway, it could enhance angiopoietin (*ANGPT*) gene expression and VEGF secretion in the luteal.

Suppression of the PROK1 signaling pathway via the receptor antagonist PC7 has been shown to significantly reduce the phosphorylation levels of MAPK1/3 and PTK2 in trophoblast cells on days 15 and 20 of pregnancy, which disrupts processes such as cell proliferation and angiogenic gene expression^30^. Tibery and colleagues found that, in women with endometriosis, PROK1 expression was significantly downregulated compared with healthy controls, suggesting that PROK1 may play a critical role in vascular function during the peri-implantation phase and early pregnancy. Variations in PROK1 levels could contribute to abnormalities in the eutopic endometrium associated with endometriosis. Previous studies have demonstrated that inhibition of the PROK1/PROKR1 pathway reduces angiogenesis and endometrial receptivity, which underscores its significance in reproductive health^31^.

On the other hand, some studies emphasize the adverse effect of PROK1-PROKR1 in pregnancy failure or outcomes. For example, Karaer et al.^32^. reported that PROK1 expression levels were increased in women with RIF compared with controls, whereas PROK1 protein levels did not differ between the two groups. However, they found that PROKR1 mRNA and protein levels were overexpressed in RIF compared with controls. They suggested that the PROK1/PROKR1 system was changed in the endometrium of women with RIF, especially *PROKR1* downregulation, which significantly affected endometrial receptivity. Furthermore, in an animal study, Reynaud et al.^33^. suggested that using an antagonist of PROKR1 (PC7) could significantly enhance trophoblast invasion; however, no significant changes were found in PROKR1-related genes, such as *Hand1*,* Mash2*, and *Pl2*.

Progesterone and its receptors (PGR) play an essential role in preparing the endometrium for successful implantation. This includes processes such as receptivity, decidualization, regulation of endometrial stromal and epithelial cells, and angiogenesis^4,5,34^. In our study, we found that *PGR-B* gene and protein levels were significantly lower in women with RIF than in fertile women. A recent survey by Al-Lamee et al.^34^. found that PGR expression was reduced across different endometrial cell types in women with RIF and recurrent pregnancy loss (RPL) when compared to fertile controls. Other research has shown that many infertile women, along with those who have endometriosis, also exhibit lower PGR expression or altered protein levels when compared to healthy fertile women^35,36^.

Additionally, Petousis et al.^35^. examined *PGR-A* and *PGR-B* expression in endometrial tissue from women with unexplained infertility and endometriosis. They noted significant downregulation in both conditions compared to healthy fertile women. Hosseinirad et al.^4^. conducted a cross-sectional study to assess *PGR* expression and its phosphorylated form in endometrial stromal cells from RIF patients compared with healthy fertile women. They found a significant reduction in *PGR* mRNA (*P* < 0.01) and phospho-Ser294 PGR protein (*P* < 0.05) levels in RIF patients. They suggested that the expression of PGR and its phosphorylated form is impaired in RIF patients. Moreover, other studies have shown that *PGR-B* expression is downregulated in women with infertility and endometriosis^37–39^. Furthermore, Dixit and colleagues found that the expression of both the *PGR* gene and protein was substantially reduced in the epithelial and stromal cells of women with infertility. They suggested that measuring PGR expression in the endometrium through immunohistochemistry could help differentiate between normal and impaired endometrial function^36^.

The proposed molecular mechanism suggested that suppression of PGR in epithelial tissues leads to a significant downregulation of uterine progesterone-responsive genes, including Indian hedgehog (*Ihh*), *HOXA10 (HOXA10)*, and leukemia inhibitory factor (*LIF*). In contrast, for example, in the PGR knock-out model, administration of recombinant LIF (rLIF) does not restore embryo attachment. This finding suggests that epithelial PGR is involved in embryo attachment through multiple pathways that extend beyond the LIF-STAT3 signaling pathway^40^.

The results indicated a relationship between the PGR-B gene and protein and the PROK1 gene and protein in the endometrial tissue of fertile women. However, this relationship was absent in women with recurrent implantation failure (RIF). Additionally, PROKR1 overexpression was shown to enhance angiogenesis and gene expression^41^. Chi and colleagues demonstrated that PGR binding to uterine chromatin can alter gene expression, thereby improving endometrial receptivity and supporting functions such as cell differentiation and angiogenesis through ANGPT2 and VEGF^41^.

Goryszewska-Szczurek et al.^44^. demonstrated that estradiol (E2) and progesterone (P4) upregulate the expression of PROK1 and PROKR1 in porcine endometrial explants, corroborating previous findings in human studies. This regulation is mediated by a steroid hormone response element located within the PROK1 promoter, which plays a crucial role in modulating gene expression and uterine receptivity^32,42,43^. Furthermore, PROK1 may interact with steroidogenic factors such as *STAR* and *CYP11A1*, potentially enhancing progesterone synthesis^30^. These findings suggest that the pathways mediated by progesterone receptors (PGR) can significantly amplify PROK1 mRNA expression, thereby advancing our understanding of the molecular mechanisms underpinning reproductive health. Additionally, the co-expression and regulatory potential between PGR-B and PROK1, as shown by GeneMANIA, underscore the relevance of these interactions for diagnostic and therapeutic strategies (https://genemania.org).

**M**icroRNAs (miRNAs) and long non-coding RNAs (lncRNAs) play crucial roles in regulating fertility and infertility by modulating gene expression. lncRNAs and miRNAs are key post-transcriptional regulators that control gene and protein expression by modulating mRNA stability and translation. The miRNAs typically repress gene expression by binding to mRNA 3′-UTRs, promoting degradation or blocking translation. In contrast, lncRNAs (longer than 200 nucleotides and not translated into protein) counteract this repression by sequestering miRNAs, masking their binding sites, interacting with RNA-binding proteins, or influencing epitranscriptomic marks like m6A. Together, these molecules fine-tune cellular function, and their dysregulation is linked to diseases such as infertility, cancer, neurodegeneration, and autoimmunity^15–19,44^.

The present study found that in patients with RIF, the expression of *lncRNA-H19* and *miR-210-5p* was significantly lower than in the control group (Fig. 1). In a cross-sectional survey, Zeng et al.^20^. found that lncRNA-H19 expression significantly reduced in RIF women compared to controls. They suggested that the association between low *lncRNA-H19* expression and integrin β3 protein was linked to impaired receptivity. Furthermore, downregulation of *lncRNA-H19* and reduced *HOXA10* expression compromised endometrial receptivity and decidualization in endometrial stromal cells, ultimately impairing embryo implantation^21^.

Zeng and colleagues further elucidated the role of lncRNA H19 in angiogenesis in extravillous trophoblasts (EVTs). They found that lncRNA-H19 targets the anti-angiogenic miRNA-106a-5p, promoting VEGF-A expression in EVTs^45^. Furthermore, lncRNA-H19 is expressed in antral and cystic atretic follicles in the ovaries, which is associated with the maturation of antral follicles. H19 is expressed in cytotrophoblasts in placental tissues and plays a crucial role in trophoblast development. Dysregulated expression of H19 has been implicated in pre-eclampsia and fetal growth restriction. Consequently, H19 is a potential biomarker and therapeutic target for various ovarian and placental pathologies^17,22^.

Co-expression of lncRNA-H19 and HOXA10 occurs early in pregnancy, with HOXA10 levels increasing to prepare the endometrium for implantation. Moreover, exposure to estradiol (E2) and progesterone (P4) significantly increases HOXA10 and HOXA11 expression. Knockouts of PGR and ESR genes in animal models result in downregulation of HOXA10 and HOXA11, indicating that steroid hormones and their receptors regulate these genes and thereby impact endometrial receptivity and decidualization. Therefore, both PGR-B and lncRNA-H19 are implicated in the upregulation of HOXA10 gene expression. This suggests that lncRNA-H19 may potentiate the effects of PGR-B through direct interactions or alternative signaling pathways. Such mechanisms could enhance decidualization and optimize endometrial receptivity^46^. The relationship between lncRNA H19 expression and pregnancy complications in the IVF context has led to conflicting results. While studies indicate that lncRNA-H19 is downregulated in murine placentas after IVF, human IVF pregnancies demonstrate an upregulation of H19. Early-onset preeclamptic patients have notably reduced H19 levels linked to hypermethylation of its promoter. These discrepancies may arise from differences across pregnancy stages and interspecies differences in placental biology. However, evidence suggests a potential loss of genomic imprinting at the H19 locus in IVF-derived placentas, which correlates with adverse pregnancy outcomes^47^.

In this study, a significant positive association was observed between miR-210-5p levels and the genes PGR-B and PROK1. It was noted that miR-210-5p expression was higher in fertile women compared to patients with Recurrent Implantation Failure (RIF). Additionally, computational analyses indicated potential interactions between miR-210-5p and both PGR-B and PROKR1, suggesting a possible regulatory network involving these components (https://www.targetscan.org/vert_72/, https://dianalab.e-ce.uth.gr/tarbasev9). Li et al.^48^. found that miR-210 expression is downregulated in trophoblasts of women with unexplained recurrent spontaneous abortion (URSA), suggesting its inhibition leads to decreased cell proliferation, increased Caspase 3 activity, and elevated Bax levels while decreasing Bcl-2. This could also reduce PI3K/Akt pathway activity and increase TNF-α and IL-6 secretion. Aoyagi et al.^49^. identified different miRNA profiles in women with normal endometrium versus those with endometriosis (EM), finding higher miR-30a-5p in normal tissues and elevated miR-210 in EM. Both miRNAs regulate IGFBP-1 and prolactin (PRL) but through distinct pathways. Increased miR-210 in EM was linked to downregulated GHR mRNA and thymidine kinase, suggesting it may induce IGFBP-1 and stimulate PRL. Research on baboon and human endometrium indicated that miR-210 suppresses IGFBP3, which is downregulated in endometriosis, potentially promoting cell proliferation and migration^50^. Additionally, miR-210 expression was higher in large follicles, impacting clinical pregnancy and endometrial receptivity^51^. Furthermore, miR-210-enriched extracellular vesicles (EVs) regulate angiogenesis by targeting genes like placental growth factor (PGF) and VEGF in HUVECs. Levels of miR-210 significantly increase from day 1 to day 4 post-implantation, supporting essential cellular functions during development^52^.

However, other Studies showed that miR-210 is linked to recurrent spontaneous abortion (RSA), inflammation, and preeclampsia (PE). For instance, Kopriva et al.^53^. found that HIF-1α and NF-κBp50 enhance miR-210 expression in mouse placentas. In human cytotrophoblasts, increased levels of these factors and miR-210 were observed. MiR-210 targets STAT6, reducing IL-4 production and promoting pro-inflammatory cytokines, suggesting its role in TLR3-induced preeclampsia. Huang et al.^54^. reported that miR-210-5p is significantly elevated in women with RSA, indicating its importance in RSA development. Overall, miR-210 affects mitochondrial dysfunction, angiogenesis, and immune response in PE^53^.

The study presents findings from bioinformatics analyses indicating that resources such as miRPathDB and TargetScan have identified target genes for miRNA-210-5p, including PGR-B, PROK1, LIF, and HOXA11, whose expression may be regulated by miR-210. Krawczynski and colleagues reported increased expression of miR-210 target genes, including key endometrial and placental genes such as HOXA1, EFNA3, NDUFA4, and ISCU, under normoxic conditions, compared with knockout mouse models. This observation suggests that miR-210 may play a key role in regulating HOXA gene expression, consistent with the functional implications of PGR-B^55,56^.

Investigating the relationship between miR-210-5p and PGR-B expression in the endometrium during early implantation is crucial for understanding molecular regulations. The findings suggest that PGR-B, PROK1, LIF, and HOXA11 may be targets of miR-210-5p, suggesting a potential regulatory pathway. However, we acknowledge that these associations are primarily based on in silico predictions and correlation analyses, and have not yet been substantiated through functional assays^46,56^. Therefore, while our data lend support to a plausible regulatory axis involving miR-210-5p and lncRNA-H19, this relationship remains hypothetical and requires further validation using methods such as RNA immunoprecipitation (RIP), luciferase reporter assays, and siRNA. Importantly, our investigation did not establish a direct correlation between miR-210 and the expression of either gene targets or lncRNA-H19. We propose that dysregulation of miR-210-5p could compromise implantation and angiogenesis, underscoring the need for further research in this area.

Taken together, the present study provides novel insight into the molecular mechanisms underlying recurrent implantation failure (RIF). To our knowledge, this is the first human study to simultaneously examine the expression and interrelationship of PGR-B, PROK1, and PROKR1 alongside their potential regulatory non-coding RNAs lncRNA-H19 and miR-210-5p within the same endometrial tissue samples. Previous studies have focused on these molecules individually in reproductive contexts—such as the role of PGR-B in endometrial receptivity or PROK1 signaling in implantation—yet none have investigated them as components of an integrated regulatory network. Our findings reveal a concurrent downregulation of these genes and their correlated expression patterns, suggesting a coordinated regulatory axis that may influence the molecular environment required for successful implantation. Furthermore, the positive associations between PGR-B, lncRNA-H19, and miR-210-5p indicate a previously unreported post-transcriptional regulatory mechanism potentially linking hormonal and angiogenic signaling pathways in endometrial receptivity. Collectively, this integrative analysis advances our understanding of the molecular interplay between progesterone signaling, prokineticin pathways, and non-coding RNA regulation in the endometrium, offering new perspectives for biomarker discovery and targeted therapeutic strategies in women with RIF.

### Limitations

A significant limitation of this study is that the conclusions drawn are primarily based on expression data, which may not fully capture the complexity of the regulatory interactions at play. This underscores the urgent need for further investigations, such as functional assays and in vivo studies, to validate these findings and establish a more comprehensive understanding. Currently, there is insufficient empirical evidence to clearly substantiate the role of microRNAs (miRNAs) in the regulatory mechanisms governing PGR-B (progesterone receptor B) and PROK1 (prokineticin 1) in the specific cell types analyzed using luciferase assays. Additional methodologies warranting further investigation include the luciferase reporter assay, transfection with miR-210 mimics and inhibitors, manipulation of lncRNA-H19 through overexpression and knockdown, and AGO2 RNA immunoprecipitation (RIP).

Additionally, the study does not examine potential downstream signaling pathways, which could shed light on the intricate molecular mechanisms that mediate the interactions between PGR, PROK1, and the miRNA-lncRNA (long non-coding RNA) networks. Investigating these pathways is essential for elucidating the broader context of their regulatory roles. Moreover, it is important to consider the potential impact of decidualization on the expression levels of the candidate genes under study. This process may directly influence the gene expression profiles, thereby confounding the results and interpretations. Overall, addressing these limitations will be crucial for advancing our understanding of the regulatory dynamics at work in this biological context.

## Conclusion

In summary, this study demonstrates that recurrent implantation failure (RIF) is associated with significant downregulation of PGR‑B, PROK1, and PROKR1, as well as altered expression of lncRNA‑H19 and miR‑210‑5p in endometrial tissue. These molecules form part of a regulatory network governing endometrial receptivity, angiogenesis, and decidualization—biological processes essential for successful embryo implantation. The observed correlations between PGR‑B, PROK1, PROKR1, lncRNA‑H19, and miR‑210‑5p suggest a disrupted interplay in RIF patients compared to fertile controls. While positive associations among these factors were evident in fertile women, the inverse correlation between miR‑210‑5p and PGR‑B in RIF highlights a novel mechanistic disruption in the molecular signaling pathways that support implantation.

This work provides new evidence of coordinated gene–noncoding RNA interactions in human endometrium, underscoring the importance of angiogenic and hormonal signaling in implantation biology. By identifying these correlations, our study adds novel insight into the molecular mechanisms underlying impaired endometrial receptivity, offering potential biomarkers and therapeutic targets for women experiencing recurrent implantation failure.

## Supplementary Information

Below is the link to the electronic supplementary material.

Supplementary Material 1

Supplementary Material 2

Supplementary Material 3

Supplementary Material 4

## Data Availability

The data that support the findings of this study are available from Iran University of Medical Sciences but restrictions apply to the availability of these data, which were used under license for the current study and therefore are not publicly available. Data are, however, available from the corresponding author upon reasonable request and with permission of ***Iran University of Medical Sciences*** , in accordance with ethical approval ( **IR.IUMS.FMD.REC.1402.040** ) and patient confidentiality regulations.
