# Supplementary material for: Progesterone receptor, prokineticin 1, LncRNA-H19, and miR-210 in molecular regulation of recurrent implantation failure
Source: Sci Rep. 2026 Jun 8;16:26068. doi: 10.1038/s41598-026-41482-7 (PMC13490531; doi:10.1038/s41598-026-41482-7)
Supplement: Supplementary file 1 [file 41598_2026_41482_MOESM4_ESM.docx]

**Supplementary Information**

**For the manuscript entitled:**

“Progesterone Receptor, Prokineticin 1, LncRNA-H19, and miR-210-5p in Molecular Regulation of Recurrent Implantation Failure”

**Supplementary Methods**

Effect sizes, Bonferroni correction, false discovery rate (FDR) adjustment, and statistical power analyses were performed using IBM SPSS Statistics (Version 24.0; IBM Corp., Armonk, NY, USA) and R software (package *'pwr*'). For non-normally distributed variables, the Mann–Whitney U test was applied and effect size was calculated as rank-biserial correlation (r). For normally distributed variables, independent-samples t-tests were used and effect size was calculated as Cohen’s d. Multiple testing correction was performed using both Bonferroni adjustment and the Benjamini–Hochberg false discovery rate (FDR) procedure. Statistical significance was defined as adjusted p < 0.05. Power analyses were conducted using observed effect sizes with α = 0.05 and n = 50 per group.

**Table S1. Power analysis for gene and protein expression comparisons** (R package pwr)

| **Marker** | **Test** | **Effect size** | **n (per group)** | **α** | **Power (1−β)** | **Interpretation** |
| --- | --- | --- | --- | --- | --- | --- |
| **PGR-B (gene)** | Mann–Whitney U (r = 0.63) | r = 0.63 | 50 | 0.05 | **>0.99** | Excellent power |
| **PROK1 (gene)** | Mann–Whitney U (r = 0.60) | r = 0.60 | 50 | 0.05 | **>0.99** | Excellent power |
| **PROKR1 (gene)** | Mann–Whitney U (r = 0.62) | r = 0.62 | 50 | 0.05 | **>0.99** | Excellent power |
| **lncRNA-H19** | Mann–Whitney U (r = 0.68) | r = 0.68 | 50 | 0.05 | **>0.99** | Excellent power |
| **miR-210** | t-test (d = 1.54) | d = 1.54 | 50 | 0.05 | **>0.99** | Excellent power |
| **PGR-B protein** | t-test (d = 4.80) | d = 4.80 | 50 | 0.05 | **≈1.00** | Complete power |
| **PROK1 protein** | t-test (d = 3.35) | d = 3.35 | 50 | 0.05 | **≈1.00** | Complete power |

**Table S2. Comparison of gene and protein expression levels between RIF patients and fertile controls**

| **Marker** | **Distribution** | **RIF (n=50)** | **Control (n=50)** | **Test** | **Effect Size** | **Raw p** | **Bonferroni p** | **FDR p** | **Significant** |
| --- | --- | --- | --- | --- | --- | --- | --- | --- | --- |
| **PGR-B (gene)** | Non-normal | 0.235 (0.01–0.567) | 1.62 (0.30–4.61) | Mann–Whitney U | r = 0.63 | <0.001 | <0.001 | <0.001 | **Yes** |
| **PROK1 (gene)** | Non-normal | 0.165 (0.03–1.34) | 0.93 (0.33–3.16) | Mann–Whitney U | r = 0.60 | <0.001 | <0.001 | <0.001 | **Yes** |
| **PROKR1 (gene)** | Non-normal | 0.20 (0.03–1.99) | 1.72 (0.34–6.11) | Mann–Whitney U | r = 0.62 | <0.001 | <0.001 | <0.001 | **Yes** |
| **lncRNA-H19** | Non-normal | 0.26 (0.03–1.23) | 1.91 (0.55–3.77) | Mann–Whitney U | r = 0.68 | <0.001 | <0.001 | <0.001 | **Yes** |
| **miR-210** | Normal | 0.89 ± 0.34 | 2.06 ± 0.96 | t-test | d = 1.54 | <0.001 | <0.001 | <0.001 | **Yes** |
| **PGR-B protein** | Normal | 0.75 ± 0.06 | 1.06 ± 0.07 | t-test | d = 4.80 | <0.001 | <0.001 | <0.001 | **Yes** |
| **PROK1 protein** | Normal | 0.75 ± 0.06 | 1.02 ± 0.10 | t-test | d = 3.35 | <0.001 | <0.001 | <0.001 | **Yes** |

**Statistical Notes:**

- **RIF**: Recurrent Implantation Failure; **Control**: Fertile women without implantation failure
- **Effect sizes**: *r* = rank-biserial correlation for Mann–Whitney U test; *d* = Cohen’s *d* for independent-samples *t*-test
- **Adjusted p-values**: Bonferroni and Benjamini–Hochberg false discovery rate (FDR) corrections were applied to control for multiple comparisons
- **Blinding and randomization**: All statistical analyses were performed under blinded conditions, and sample processing order was randomized to minimize bias

**Table S3. Correlation analysis between gene and non-coding RNA expression levels**

| **Variable 1** | **Variable 2** | **r-value** | **p-value** | **FDR-adjusted p-value** | **Group** |
| --- | --- | --- | --- | --- | --- |
| PGR-B | PROK1 | 0.425 | 0.0000 | **0.0000** | RIF |
| PGR-B | lncRNA-H19 | 0.356 | 0.011 | **0.0151** | RIF |
| PROK1 | lncRNA-H19 | 0.428 | 0.002 | **0.0046** | RIF |
| PGR-B | miR-210-5p | −0.293 | 0.039 | **0.0390** | RIF |
| PGR-B | PROK1 | 0.552 | 0.0000 | **0.0000** | Control |
| PROK1 | PROKR1 | 0.354 | 0.011 | **0.0151** | Control |
| PGR-B | PROKR1 | 0.319 | 0.019 | **0.0232** | Control |
| PGR-B | miR-210-5p | 0.317 | 0.0024 | **0.0046** | Control |
| PROK1 | miR-210-5p | 0.449 | 0.001 | **0.0037** | Control |
| PGR-B | lncRNA-H19 | 0.305 | 0.0312 | **0.0343** | Control |
| PROK1 | lncRNA-H19 | 0.419 | 0.0025 | **0.0046** | Control |

*) This table summarizes the pairwise correlations between PGR-B, PROK1, PROKR1, lncRNA-H19, and miR-210-5p expression levels in both study groups. Spearman’s rank correlation coefficients (r-values) and raw p-values are reported, alongside FDR-adjusted p-values calculated using the Benjamini–Hochberg procedure to correct for multiple comparisons. Statistically significant associations (FDR-adjusted p < 0.05) indicate robust molecular relationships, particularly among PGR-B, PROK1, and non-coding RNAs in fertile women, and a disrupted regulatory pattern in RIF patients.

**Figure S1. Proposed mechanistic model illustrating hypothesized molecular interactions involved in endometrial receptivity and recurrent implantation failure (RIF).**

**Figure S2. Coexpression, physical interaction, and downstream gene relationships concerning PGR, PROK1, and PROKR1 are examined in the context of implantation and endometrial receptivity form GeneMania.**
